# Engaging critically: exploring the varying roles of lived experience advisors in an implementation science study on management of opioid prescribing

**DOI:** 10.1186/s40900-024-00552-8

**Published:** 2024-02-09

**Authors:** Emily Nicholas Angl, Celia Laur, Michael Strange, Barbara Sklar, Mina Tadrous, Noah Ivers

**Affiliations:** 1 ENA Consulting, Toronto, ON Canada; 2 Women’s College Hospital Institute for Health System Solutions and Virtual Care, 76 Grenville Street, Toronto, ON Canada; 3 Institute of Health Policy, Management and Evaluation, University of Toronto, Health Sciences Building, 155 College Street, Suite 425, Toronto, ON Canada; 4 Lived Experience Advisor, Toronto, ON Canada; 5 Leslie Dan Faculty of Pharmacy, University of Toronto, 144 College Street, Toronto, ON Canada

**Keywords:** Patient engagement, Implementation science, Patient oriented research

## Abstract

Involvement of individuals with lived experience, also called “patient partners”, is a key element within implementation science, the study of how to put evidence into practice. While conducting a 4-year implementation study focused on improving physician management of opioid prescribing, our research team worked closely with Lived Experience Advisors (LEAs). LEAs were involved throughout the study, including developing patient-facing recruitment material, informing the analysis of results, and as a regular reminder of the real-world impact of this work. However, through regular critical reflection, we acknowledged that we were still uncertain how to articulate the impact of LEA involvement. As a team, we continually discussed why and how people with lived experience were involved in this study. We probed ill-defined concepts such as “patient perspective”, which was particularly complex for a study focused on changing physician behaviour with indirect impact on patients. This critical reflection strengthened trust and rapport between team members (characteristics deemed essential to meaningful patient involvement), while underscoring the value of including concerted time to explore the muddier aspects of engagement. In short, patient engagement did not proceed as smoothly as planned. We advocate that “best practices” in the engagement of people with lived experience include regularly setting aside time outside of practical study tasks to interrogate complex aspects of patient engagement, including reflecting on how and why individuals with lived experience are involved.

## Abstract (plain language summary)

Involvement of individuals with lived experience, also called “patient partners”, is often a required element of applied research. Although there is a lot of guidance on how to engage individuals with lived experience, there is no single best-practice that always applies. Each team is different and must adapt to meet the needs of their study and team. While conducting a 4-year study focused on improving physician management of opioid prescribing, our research team worked closely with Lived Experience Advisors (LEAs). The LEAs were involved in developing patient-facing recruitment material, informing the analysis of results, and were a regular reminder of the real-world impact of this work. As a team, we continually discussed why and how individuals with lived experience were involved in this study and probed concepts such as “patient perspective”, which is complex in a study focused on changing physician behaviour. Setting aside time to not just work on a task but to critically reflect and ask questions led to new insights into why and how we do this work. For example, one of the patient handouts that was co-designed with patients and praised by some physicians we interviewed, was found by LEAs to be objectifying and lacking nuance, which further highlighted how the same material can be received in different ways. Our discussions also helped build trust and rapport, which are characteristics deemed essential to meaningful patient involvement. We advocate for study teams to dedicate time to interrogate the less straightforward aspects of patient engagement. In other words - “embrace the messiness”.

## Introduction

Implementation science is the study of methods and strategies that help put research evidence into practice [1]. By design, teams conducting research where a new program or process is being implemented in a healthcare setting, include a variety of perspectives [2]. While team members in these applied studies often come from a range of academic and professional backgrounds, patient and community members had previously only been included as research subjects [3]. Today, it is increasingly common to involve people with ‘lived experience’ in implementation science studies, alongside more traditional roles such as clinician or statistician [1, 4]. While personal experience certainly has influence, broadly speaking, these latter roles are associated with a defined body of knowledge and specific training. Alternatively, a person bringing a patient or public voice to the research team is expected to draw on much more than their interactions with the healthcare system. Their input and advice is understood to be impacted by the entirety of their experience—their culture, education, family and friends, and, for some patient partners, by other research studies or committees they have been involved in. Incorporating such diverse perspectives into a research system which is built around expertise from formal education, adds to the complexity of implementation science studies, while also adding value by providing a deeper understanding of the real-world impact of the topics being studied.

This Comment discusses a case study of how our team of researchers and individuals with lived experience worked, and reflected, together during a 4-year study focused on improving physician management of opioid prescribing. The topics, ideas, and quotations presented in this Comment are drawn from discussions during our team ‘check-ins’ and 1-on-1 meetings, as well as our continual reflections. Details about the overall study methods and results are published separately [5, 6].

## The study

“As this study was presented as a way to help doctors perform better pain management to patients, I was—and remain—both flattered and honoured to be asked my thoughts and opinions.” (Lived Experience Advisor; author).

This team was brought together through a Canadian Institutes of Health Research (CIHR), Strategy for Patient Oriented Research (SPOR)-funded study. This implementation science study aimed to understand how two pre-existing, large-scale interventions supported safer opioid prescribing by physicians across Ontario, Canada, and how they can be optimized. In one intervention, led by Ontario Health, the provincial advisor on quality in healthcare, primary care physicians were provided with individualized data about their prescribing including how they compared to a standard or target, and changes over time [7]. Although the report provided data on several prescribing practices, our focus was on the section of the report specific to opioid prescribing. The second intervention was an academic detailing service (also known as educational outreach), led by the Centre for Effective Practice, an independent not-for-profit organization supporting quality improvement in primary care [8]. In the academic detailing intervention, pharmacists conducted a series of visits with primary care physicians to discuss their opioid prescribing practices and provide resources to support improvements in care delivery.

The study included two highly technical analyses: (1) a quantitative analysis to quantify trends of provincial-level administrative data to show the impact of the two interventions; [9]; (2) a qualitative analysis which applied advanced behaviour change theory to understand fidelity from the intervention-design through to the patient-encounter [5, 6]. This qualitative analysis included mapping relevant documents (the report delivered by Ontario Health, and the training resources provided by the pharmacists) to specific behaviour change techniques [10], and deductive thematic analysis of interviews with intervention leads/designers, physicians who received the interventions, and patients of those physicians [5, 6]. Interviews were planned for patients of physicians who had received both interventions and been interviewed. However, our recruitment of physicians was lower than anticipated and most physicians were unable to recruit their patients, largely due to the additional burden caused by the COVID-19 pandemic. All recruitment and interview materials were prepared for these patient interviews, and ethics approval received, however this part of the study had to be cancelled due to lack of eligible patients. Conducting these patient interviews would have been an obvious place for engaging persons with lived experience in the research process. When these patient interviews were cancelled, this raised further questions regarding how to ensure value for, and from, patient partners. In other words, it was important to the team at the outset to demonstrate exactly how patient contributions made a difference to the study, but the opportunities to do this shifted over time.

Adding to the complexity of the study and the engagement process, the funding was for two parallel streams of work—one on opioid prescribing (the focus of this Comment), and the other on antibiotic prescribing. From the start we knew navigating this patient-oriented study, with its parallel streams of work, advanced quantitative and qualitative analyses, and a focus on changing physician prescribing practices, would lead to many difficult questions regarding how to engage all members of our team in a way that would meet all our needs. Questions were raised, such as: What is the role of a patient partner in complex quantitative analysis when using administrative data with variables that cannot be changed? What are the most useful ways for patient partners to be involved when the study is solely focused on changing physician behaviour?

## The team

“Although, initially I was confident that personal experience with the subject matter was paramount to the issue at hand, (I originally was advising on the antibiotic side of the project), I quickly realized I could also speak to the issue as a concerned citizen who has seen and heard about the complexities of opioids over-prescribing through experiences of friends and family, as well as drawing from my professional side as a retired RN [Registered Nurse] /Educator.” (Lived Experience Advisor; author).

The research team included implementation scientists, health services researchers, health psychologists, and biostatisticians/epidemiologists. The grant was co-led by NI, a clinician scientist, and ENA, a Patient and Community Engagement Consultant with experience facilitating and researching patient and public engagement, and lived experience with opioid prescriptions. Through the grant, ENA was hired on contract to act as a bridge between patients and the research team, informing all patient and community engagement decisions. ENA was also due to conduct the patient interviews. MT and CL are researchers who led the quantitative and qualitative investigations, respectively.

Shortly after funding was announced, two individuals (BS and MS) were recruited to the team based on their personal experiences with the health system to act as “patient partners”. At the beginning, the patients engaged in the antibiotic or opioid stream of work based on their particular health experience. However, the patient engagement approaches quickly became integrated across both streams. This integration occurred for practical reasons (i.e., many topics and questions were shared between streams, and the same person led all engagement), and due to team member interest. The composition of this group and the integration of the antibiotic and opioid streams of work, prompted further discussion about what experiences were necessary and relevant for our patient partners and whether we were focused on their patient experience or looking for a more generalized public/lay perspective. Although we could not reach definitive answers to such difficult questions, probing the concept of what we meant by “patient voice” and “lived experience” helped us clarify expectations for team members. These discussions also made us think carefully about how achievable ideals such as “shared power” or “equal partners” were in a context where only a subset of the team shared very personal experiences. We acknowledged these tensions and, mainly directed by ENA, continually made sure the team was comfortable and that these power dynamics were acknowledged. A patient engagement evaluation survey was adapted and distributed 1 year into the study, however with a small team, responses could not be anonymous, and everyone felt that ongoing critical discussion was more valuable than the survey, and it was not attempted again [11].

## Processes of engagement

In the early stages, introductions were made, training was conducted, a Terms of Reference co-developed, and many discussions were had about how to approach patient engagement in this health-system focused, physician-facing work. Team members MS and BS, respectively, brought on for the opioid and antibiotics streams of work, met together. Meetings started off as bi-monthly and in-person, but shifted to being virtual, and as needed. A flexible agenda developed by ENA in consultation with the research team was developed for each meeting, including study updates, researcher requests for input, and an open invitation to get more involved in any aspect(s) of the work. Patient partners were invited to attend any study meeting, and they decided not to attend the day-to-day logistics meetings, but would be invited to attend the more summative and influential meetings, such as those with our partners (Ontario Health etc.). To make sure everyone was informed, and that all information was transparent, we created a regularly (monthly) updated tracking document and shared folder which ENA managed and was easily accessible to all. The tracking document summarized meetings, project progress, upcoming meeting dates, and links of interest, while the shared folder held key project materials, training modules and references.

As the comfort level within the team increased, meetings became more flexible and friendly with everyone sharing more personal updates, commentary on how opioid prescribing was portrayed in the media, and a frank discussion about if and how this group was actually impacting the research. It was made clear that it was not necessary for patient partners to share their personal health experiences, as this was not their role in the study (they were not research “participants”), and their contribution was determined by their comfort level and interest. However, both patient partners and ENA chose to share personal experiences. These experiences were kept confidential, and ENA followed-up with each team member when a particularly difficult experience was shared. Sharing of these experiences were not “data”, but team members indicated formally in the evaluation, and informally in team discussions, that sharing of these experiences impacted the way they viewed this work, and was a constant reminder, for researchers in particular, of the ultimate goal of improving patient care.

## Varying roles

“I constantly sat with the discomfort of not being able to clearly define what the lived experience advisors were there to represent. It felt impossible to reconcile that, although we had stated that these advisors were not to be considered the voice of all patients, there was an expectation that their involvement was making the project more patient-centred and that we were extrapolating from their input.” (Patient and Community Engagement Consultant; author).

During the early stages of the work, although the team recognized the study funder (the Canadian Institutes of Health Research) defined “patient” as an overarching term inclusive of all lived experience with a health issue, including informal caregivers [12], MS, BS, and ENA decided that the title “patient partner” did not represent how they felt about their role in this study. This feeling has also been described in other studies, with “patient” feeling too vague to accurately represent their input [13]. Instead of being referred to as a “patient”, MS and BS decided to use the term “lived experience advisors” (LEA). This shift in terminology was the result of multiple conversations regarding the difficulty in precisely defining the role of a “patient partner” and accurately describing which experiences and knowledge they draw upon. The team recognized that the LEA’s were not solely drawing on their experiences as patients, nor were they there to represent all patients. Discussions with LEAs also included questions that didn’t necessarily apply to their healthcare experience, as also reported elsewhere [14, 15].

When contributing to the development of the patient interview materials, engagement was more straightforward, including reviewing recruitment materials and interview questions. However, when this aspect of the study was cancelled, there was a shift, and in many ways, the input of the LEAs seemed more accurately described as a ‘non-researcher’ or as an ‘outside’ perspective, which led to questions and ideas about their role in a study focused on changing physician behaviour. Considering what the LEA voice represented became an important topic as we made decisions as a team. We struggled to articulate how our discussions were contributing to the study design and findings, particularly given that the interventions focused on changing physician behaviour. There are some instances when LEA opinions contributed more directly to interpretation of results. When LEAs saw some tools for physicians to use with their patients, one of the tools, which had been co-designed with patient input and praised by physicians, was seen by LEAs to be objectifying and lacking nuance. This difference of opinion highlighted that even within material developed with patients, opinions can still differ greatly in how a tool is perceived.

Initially the team created a template form to complete after meetings to capture the input of the LEA’s, the process of decision making, and the eventual impact. This type of record keeping was effective for direct input to developing recruitment material, but could not capture the less tangible outcomes, particularly for the occasions where LEA shared their experiences. The team discussed how such contributions seemed to have an impact in shaping discussions and decisions, but that it was difficult to describe or track.

Ongoing critical reflection on how the LEAs would be included highlighted that their level of involvement would vary over time and emphasized that they were not expected to represent all aspects of the “patient perspective”. We recognized that sharing personal, emotional, experiences—particularly when the purpose and impact of sharing those experiences is unclear—was fraught with challenges. As a team, we discussed the potentially significant repercussions of revisiting difficult experiences. When these personal experiences were shared, it informed our deeper understanding of lived experience with an opioid prescription, but it was agreed that this was not “data collection”. As a result of these conversations, and with the understanding that inclusion of two patient members was not sufficient, we expanded our engagement approach to include multiple strategies [16]. To begin, we reviewed previous engagement and advocacy work on similar topics, such as papers which had engaged with people with lived experience or researched their perspective. The aim of this informal review was not meant to provide a comprehensive analysis of patient perspectives on opioid prescribing in primary care. We used this literature to inform our language-use around opioids, as well as our engagement approaches to support development of our interview guides, and other engagement opportunities. We consulted with the Ontario Drug Policy Research Network (ODPRN) Citizens’ Panel three times throughout the study, particularly to inform the primary and secondary outcome measures in our quantitative analysis. We also had multiple individual meetings with two members of the ODPRN's Lived Experience Advisory Group (LEAG), a group whose members have experienced or are experiencing opioid-use disorder [17]. LEAG members informed our approach to patient interviews and piloted our interview guide. As mentioned, patient interviews were originally meant to be a key aspect of this study.

## Where does patient engagement fit?

“I constantly tried to balance how much detail and background to provide, aiming for the “sweet spot” of informative and interesting, yet not overwhelming.” (Researcher; author).

A significant challenge for our team was deciding how much input to seek from LEAs for these physician-facing interventions and identifying where LEA input could be most useful. At the same time, a core principle of patient engagement is to involve LEA members throughout. We discussed these tensions in an attempt to find the ‘just right’ amount of engagement. We explored how much theory and background was needed to provide context to the results, how to meet the interest of the LEAs while working within their capacity, and how to do everything within the available time and budget. For example, while LEAs were interested in the background of behaviour change theory, they didn’t necessarily want extensive training on the subject. They also didn’t feel the need to be involved in the actual quantitative analysis, but did want to help determine outcomes of interest in conjunction with the ODPRN Citizen's Panel.

While our responsive and reflective approach *felt* like it worked well for our team, it is important to consider the potential limitations and repercussions of such flexibility. For example, the LEA’s and ODPRN Citizen's Panel involvement in quantitative outcome selection was inherently limited since the study could only use existing administrative datasets. We also critically considered how different engagement could have been if two different individuals, with their own interests and experiences, had been on the team. As mentioned in the example above, the same patient-facing tool can be seen as beneficial for some and objectifying for others. The way that LEA’s want to be involved also does not just impact the study team, it has potential effects on those to whom the research is extrapolated. The ‘figure it out as you go’ approach is not necessarily benign. There can be profound repercussions of feeling ‘token’ in a process or feeling unheard, and judged, particularly if these feelings were felt during previous experiences within the healthcare system. All of this reinforces our call to action for concerted, critical reflection on patient engagement throughout all studies.

Further reflections on lived experience engagement within implementation science studies where patients are not the primary audience of the intervention, are presented in an episode in the Matters of Engagement podcast [18]. The iterative nature of writing this article, and developing a podcast, deepened our reflective process. We encourage others to share their experiences in a variety of ways to improve your skills in reflective practice, and so others can learn from your experience.

## Conclusion

“I found that being able to say ‘This is what I've experienced, this is what I've discovered, this is what I've heard, this is what I've seen. Not that it's right. Not that it's wrong. But that's my perception of what's happened to me’—maybe that can help somebody else. That's kind of what it boils down to, just being able to share. That was the whole real reason for me trying to get involved in this. And it still is.” (Lived Experience Advisor; author).

There are many reasons to include LEAs in implementation science studies. Ultimately, we advocate that "best practices" in the engagement of LEAs should include a concerted effort to challenge assumptions about these roles, and to evaluate where and how value is added. Talking honestly about the challenges may help those involved in patient-oriented research and patient engagement to have a better understanding of how and when to engage. Exploring these potentially uncomfortable questions together was how we developed trust and insight, characteristics deemed essential to meaningful patient involvement [19]. For us, this critical reflection positively impacted our team dynamics and was a constant reminder of the impact of this work on real people. Dedicating time to discuss the difficult questions together acknowledges the complexity and the potential effects on those impacted by the research, while also supporting learning and capacity building within the still developing field of patient engagement in research.

## Data Availability

Data sharing is not applicable to this article as no datasets were generated or analysed during the current study.

## Abbreviations

CIHR: Canadian Institutes of Health Research
LEA: Lived Experience Advisor
SPOR: Strategy for Patient Oriented Research
